# Traffic-related ultrafine particles impair mitochondrial functions in human olfactory mucosa cells – Implications for Alzheimer's disease

**DOI:** 10.1016/j.redox.2024.103272

**Published:** 2024-07-19

**Authors:** Laura Mussalo, Riikka Lampinen, Simone Avesani, Táňa Závodná, Zdeněk Krejčík, Juho Kalapudas, Elina Penttilä, Heikki Löppönen, Anne M. Koivisto, Tarja Malm, Jan Topinka, Rosalba Giugno, Pasi Jalava, Katja M. Kanninen

**Affiliations:** a A. I. Virtanen Institute for Molecular Sciences, University of Eastern Finland, 70210, Kuopio, Finland; b Department of Computer Science, University of Verona, 37134, Verona, Italy; c Department of Toxicology and Molecular Epidemiology, Institute of Experimental Medicine of the Czech Academy of Sciences, Videnska 1083, 142 20, Prague, Czech Republic; d Department of Neurology, Neuro Centre, Kuopio University Hospital, 70210, Kuopio, Finland; e Department of Otorhinolaryngology, University of Eastern Finland and Kuopio University Hospital, 70210, Kuopio, Finland; f Brain Research Unit, Department of Neurology, School of Medicine, University of Eastern Finland, 70210, Kuopio, Finland; g Department of Neurology and Geriatrics, Helsinki University Hospital and Neurosciences, Faculty of Medicine, University of Helsinki, 00014, Helsinki, Finland; h Inhalation Toxicology Laboratory, Department of Environmental and Biological Sciences, University of Eastern Finland, 70211, Kuopio, Finland

**Keywords:** Olfactory mucosa (OM), Ultrafine particles (UFP), Mitochondrial dysfunction, Oxidative phosphorylation (OXPHOS), Redox balance, RNA sequencing (RNA-Seq)

## Abstract

Constituents of air pollution, the ultrafine particles (UFP) with a diameter of ≤0.1 μm, are considerably related to traffic emissions. Several studies link air pollution to Alzheimer's disease (AD), yet the exact relationship between the two remains poorly understood. Mitochondria are known targets of environmental toxicants, and their dysfunction is associated with neurodegenerative diseases. The olfactory mucosa (OM), located at the rooftop of the nasal cavity, is directly exposed to the environment and in contact with the brain. Mounting evidence suggests that the UFPs can impact the brain directly through the olfactory tract. By using primary human OM cultures established from nasal biopsies of cognitively healthy controls and individuals diagnosed with AD, we aimed to decipher the effects of traffic-related UFPs on mitochondria. The UFP samples were collected from the exhausts of a modern heavy-duty diesel engine (HDE) without aftertreatment systems, run with renewable diesel (A0) and petroleum diesel (A20), and from an engine of a 2019 model diesel passenger car (DI-E6d) equipped with state-of-the-art aftertreatment devices and run with renewable diesel (Euro6). OM cells were exposed to three different UFPs for 24-h and 72-h, after which cellular processes were assessed on the functional and transcriptomic levels. Our results show that UFPs impair mitochondrial functions in primary human OM cells by hampering oxidative phosphorylation (OXPHOS) and redox balance, and the responses of AD cells differ from cognitively healthy controls. RNA-Seq and IPA® revealed inhibition of OXPHOS and mitochondrial dysfunction in response to UFPs A0 and A20. Functional validation confirmed that A0 and A20 impair cellular respiration, decrease ATP levels, and disturb redox balance by altering NAD and glutathione metabolism, leading to increased ROS and oxidative stress. RNA-Seq and functional assessment revealed the presence of AD-related alterations in human OM cells and that different fuels and engine technologies elicit differential effects.

## Highlights

• UFPs impair mitochondrial functions in human olfactory mucosa (OM) cells *in vitro.*
• Oxidative phosphorylation is strongly inhibited by UFP exposure in OM cells.
• UFPs disturb redox balance in OM cells by several mechanisms.
• UFP exposure disturbs antioxidant defense mechanisms leading to oxidative stress.
• Results revealed AD-specific alterations in OM cells upon exposure to UFPs.

## Graphical abstract

**Figure:**
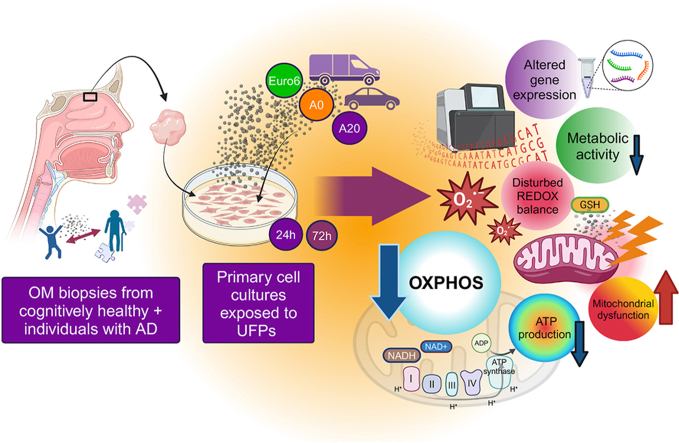

## 1 Introduction

Air pollution forms a major global burden to different aspects of health and a growing body of evidence connects it to cognitive decline as well as to the etiology of neurodegenerative diseases, including Alzheimer's disease (AD) [[1], [2], [3], [4], [5], [6], [7], [8]]. The Lancet Commission has included air pollution as a modifiable risk factor for dementia [9], and recently, the accumulation of black carbon particles in AD brain was demonstrated [10]. Ultrafine particles (UFP; diameter ≤100 nm) derived mainly from traffic emissions, are considered especially harmful due to their small size and ability to cross bodily membranes, potentially even reaching the brain [11,12]. The connection of UFPs to AD-related changes in the brain, such as increased amyloid-beta levels, perturbed redox balance, inflammation, neurotoxicity, and cognitive decline have been described in animal models [[13], [14], [15], [16], [17]]. Still, the exact mechanisms of how UFPs contribute to cellular and molecular changes in the human brain leading to AD remain obscure.

Mitochondria are one of the major targets of environmental toxicants, which can damage their morphology, function, and DNA [18]. Dysfunctional mitochondria play a critical role in the development and progression of neurodegenerative diseases such as AD. Dysfunction of mitochondria leads to excess formation of reactive oxygen species (ROS), oxidative stress, impaired oxidative phosphorylation (OXPHOS), and decreased ATP levels, leading to neurological sequelae [19,20]. In animal models, exposure to air pollutants has been linked to impairments in mitochondria, such as changes in morphology, depletion of mitochondrial DNA, increased oxidative stress, dysregulation of OXPHOS-related genes, and reduction of ATP [13,21,22]. Exposure to air pollutants is also linked with increased oxidative stress in the mitochondria of skin keratinocytes [23] and cells of the olfactory mucosa (OM) [24]. However, very little is known about how exposure to traffic-derived UFPs affects mitochondria.

The OM, located at the rooftop of the nasal cavity, is directly exposed to the inhaled air from the environment and in contact with the brain via the olfactory nerve [25]. Several studies demonstrate that the brain is targeted by pollutants via the olfactory route [1,10,[26], [27], [28]] and the connection between cognitive impairment and nasal administration of PM_2.5_ (particulate matter with a diameter smaller than 2,5 μm) has recently been demonstrated in mice [29]. Despite this evidence, the connection between UFP exposure and mitochondrial function in the context of AD has not been previously investigated in the human OM.

Therefore, by utilizing a physiologically relevant human-based *in-vitro* model of OM derived from cognitively healthy and AD donors, this study aimed to understand how exposure to different traffic-related UFPs affects the functions of mitochondria. Given that impaired olfactory identification is associated with neurodegenerative diseases such as AD, coupled with the existing knowledge that disease-associated pathological hallmarks (amyloid-beta, tau) in the olfactory bulb of AD patients correlate with the severity of AD [[30], [31], [32]], we believe our cell model fits this purpose. Previous studies with the same OM cell model from individuals diagnosed with 10.13039/100020014AD revealed disease-associated hallmarks both in transcriptomic and functional levels, further supporting the suitability of the model to investigate AD-related alterations at the human OM [[33], [34], [35]]. In our previous work, we combined fully characterized UFP exposures to OM cells derived from cognitively healthy controls and assessed molecular level alterations by RNA-Sequencing (RNA-Seq) and functional validation, and found alterations in genes related to inflammatory response, xenobiotic metabolism, olfactory signaling, and epithelial integrity [36]. In this study, we utilized previously generated RNA-Seq data from cognitively healthy controls to combine it with newly generated RNA-Seq data from individuals diagnosed with AD to compare the responses between the two groups with different health statuses. We aim to investigate molecular alterations caused by different UFPs on the mitochondria in cells derived from cognitively healthy individuals and those with diagnosed AD.

## 2 Materials and methods

### 2.1 Cell culture

Primary human OM cell cultures from n = 6 individuals diagnosed with AD were established as described in detail by Ref. [33] with ethical approval of the Research Ethics Committee of the Northern Savo Hospital District (permit number 536/2017). Written informed consent was obtained from all the donors or, for those with substantial cognitive impairment, from a family member or other proxy. In brief, OM biopsies were received via the Department of Otorhinolaryngology, Kuopio University Hospital. A biopsy of the OM was taken from the nasal septum close to the rooftop of the nasal cavity. The tissue piece was treated mechanically and enzymatically to obtain dissociated single cells and grow primary cell cultures. In experiments were used primary lines between passages 4 and 7. Cells were cultured as submerged cultures in Dulbecco's Modified Eagle Medium: Nutrient Mixture F12 (DMEM/F-12) (Gibco #11320-033) containing 10 % heat-inactivated fetal bovine serum (FBS) (Gibco #10500-064) and 1 % of penicillin-streptomycin (10,000 U/mL) (Gibco #15140-122) at humidified conditions at 37 °C, 5 % CO_2_.

### 2.2 UFP sample collection and characterization

UFP sample collection and characterization have been described in detail in our previous study [36]. In brief, the particle samples were collected by VTT Technical Research Centre of Finland from the exhausts of a modern heavy-duty diesel engine (HDE) without any aftertreatment devices and from an engine from a 2019 model diesel passenger car (DI-E6d) equipped with state-of-the-art aftertreatment devices. The HDE was run with renewable diesel (A0) and petroleum diesel (A20), whereas DI-E6d only with renewable diesel (Euro6) (Supplementary Fig. 1). Particles were collected on primary pre-treated fluorocarbon membrane filters, 70 mm (o.d.) (Fluoropore, Merck KGaA, Darmstadt, Germany). From the filters, the particles were extracted with HPLC-grade methanol, measured, and dried, and the remaining dry samples were stored at −20 °C until the cell exposures. Chemical analyses were performed to characterize the contents of metals, polycyclic aromatic hydrocarbons (PAHs), nitrogen compounds (NO_x_), organic carbon (OC), and elemental carbon (EC) in the collected samples. In addition, particle size distribution quantification revealed all samples as UFPs with the majority of particles being ≤0.1 μm by an aerodynamic diameter. Characterization revealed remarkable differences between the HDE-derived samples compared to the DI-E6d sample. A0 and A20 emissions had larger amounts of the smallest particles, as well as high levels of NO_x_ and a wide repertoire of different PAHs in comparison to the Euro6.

### 2.3 UFP exposures

UFP exposures were carried out as previously described [36]. Briefly, UFP samples were prepared by suspending dry particles in 10 % v/v sterile Dimethylsulfoxide (DMSO) (Sigma-Aldrich #D2650) in sterile water (Baxter) and sonicating in an ultrasonic water bath for 30 min. To rule out the effect of DMSO per se, treatments were compared to corresponding vehicle (10 % v/v sterile DMSO in sterile water) -treated cells. Cells were exposed to pollutants in a final concentration of 20 l/ml in a culture medium. As the dose metric, the volume refers to the amount of exhaust run through the collection filter. Mass is known to correlate poorly with the surface area of UFPs [37], and especially when using extremely clean emissions with hardly any particles in them, therefore comparison of emissions in this study is more valid by using volumetric doses (see Supplementary Table 1 UFP volume conversion to mass). After exposure, cells were incubated for 24-h, or 72-h at humidified conditions at 37 °C, 5 % CO_2_.

### 2.4 Measurement of metabolic activity and cytotoxicity

MTT (3-(4.5-dimethylthiazol-2-yl)-2.5-diphenyltetrazolium bromide) tetrazolium reduction assay was used to assess the metabolic activity of UFP-exposed cells. After the exposure to UFPs for 24-h and 72-h, cells were incubated in a medium containing 1.2 mM thiazolyl blue tetrazolium bromide (VWR #0793-1G) at 37 °C for 2.5 - 3-h. After incubation, the media was removed and DMSO (Fisher Chemical, D/4121/PB15, 67-68-5) was added to the cells to solubilize the salt. The absorbances were read at 595 nm with a Wallac Victor 1420 plate reader (PerkinElmer, Waltham, USA), and the background signal (measured with DMSO) was subtracted from readings. All values were normalized to vehicle-treated cells and as negative control was used cells lysed with Triton-X 100 (Sigma-Aldrich, 9002-93-1). Results are presented as a mean from four technical replicates/conditions/cell lines. Lactate dehydrogenase (LDH) release was quantified from collected culture media with CyQUANT™ LDH Cytotoxicity Assay Kit (#C20301 Invitrogen) according to the manufacturer's instructions. Absorbances were read at 490 nm and 650 nm using the Wallac Victor 1420 plate reader (PerkinElmer, Waltham, USA). Cells lysed with Triton-X 100 served as a positive control for cell death. Cytotoxicity is presented as a percentage of absolute cell death.

### 2.5 Transcriptomic analysis

RNA-seq was performed as previously described [36]. Briefly, RNA was extracted from six primary OM cell lines using kit AllPrep DNA/RNA/miRNA (Qiagen # 80224), DNase treatment included. The RNA quality was determined using the RNA Pico or Nano 6000 LabChip kit (#5067-1513 or # 5067-1511 Agilent Technologies, USA) and run in an Agilent 2100 Bioanalyzer. Samples selected for sequencing had RNA integrity numbers higher than 6. RNA yield was analyzed using Qubit 4 Fluorometer and Qubit™ RNA High Sensitivity (HS) assay (Invitrogen). NEBNext® rRNA Depletion Kit v2 (Human/Mouse/Rat) (New England BioLabs) was used to deplete ribosomal RNA. NEBNext® Ultra™ II Directional RNA Library Prep Kit for Illumina® (New England BioLabs) was used to prepare cDNA libraries. Purification and size selection were employed with SPRi beads (Beckman Coulter Inc.). Sequencing was performed as paired-end on the Illumina NovaSeq 6000 platform using NovaSeq 6000 S1 Reagent Kit v1.5 (200 cycles) (Illumina).

RNA-sequencing analysis was performed separately on 24-h and 72-h exposed AD samples following the same procedure previously described [36]. In summary, sequencing data were preprocessed using the Trimmomatic tool [38], aligned to the reference genome using STAR [39] and the reads quantification was performed using FeatureCounts [40]. DESeq2 R package [41]. was used to perform differential expression analysis. Exploratory analysis detected a notable batch effect attributed to the cell line of origin of each sample which was appropriately addressed in the statistical analysis. Additionally, at the 72-h timepoint, two samples exposed to Euro6 were excluded from the analysis since they behaved as outliers. Data were balanced not considering in the statistical analysis genes not expressed in at least one sample for each sample group. Differential expression analysis was performed to compare the gene expression profile of each exposure group with the relative vehicle group and genes with an adjusted (Benjamini-Hochberg) p-value <0.05 were kept as significant. These significant genes were further investigated through QIAGEN IPA [42,43] functional analysis.

### 2.6 Assessment of oxygen consumption rate (OCR)

For mitochondrial respiration assessment, the Seahorse XF Cell Mito Stress Test Kit (Agilent #102416-100) was used according to the manufacturer's instructions. The OM cells were seeded on cell XFe96-well culture microplates with a density of 15,000 cells per well. Cell culture media containing UFPs were removed, and cells were washed once with the Seahorse XF DMEM medium pH 7.4 (Agilent #103575-100) supplemented with 25 mM d-glucose (Sigma-Aldrich #G8769), 2 mM sodium pyruvate (Gibco #11360070), 2 mM GlutaMAX (Gibco #35050061). Cells were incubated at 37 °C in the absence of CO_2_ for 1 h. Oxygen consumption rates (OCR) were measured using the Seahorse XFe96 analyzer (Agilent). Baseline OCR measurement was followed by administration of an ATP synthase inhibitor (oligomycin, 1 μM), an uncoupler of oxidative phosphorylation (FCCP, 1 μM), and a mix of complex I and complex III inhibitors (rotenone and antimycin A, final concentration 1 μM). OCR measurements were normalized against the total protein and an average of two to five technical replicates per exposure was calculated. If the cells were observed to be detached around the sensors before the measurement or values were negative, wells were excluded from the analysis. The following respiratory parameters of our interest were achieved with the Seahorse Wave software (Agilent): basal respiration: OCR before the addition of oligomycin, proton leak: OCR after the addition of oligomycin, maximal respiration: OCR after the addition of FCCP, spare respiratory capacity: maximal OCR – baseline OCR, coupling efficiency: (baseline OCR - OCR after the addition of oligomycin)/baseline OCR and Non-Mitochondrial Oxygen Consumption: the minimum rate of OCR after addition of rotenone & antimycin A. Results shown are OCR values presented as a mean calculated from two tofive technical replicates.

### 2.7 Oxidative stress

The cell-permeant 2′,7′-dichlorodihydrofluorescein diacetate (H_2_DCFDA) (Thermo #D399) was used to assess intracellular oxidative stress of UFP-exposed OM-cells. Nonfluorescent H_2_DCFDA is converted to the highly fluorescent 2′,7′-dichlorofluorescein (DCF) by oxidation and/or cleavage of the acetate groups by esterases, which is indicative of the total ROS levels and oxidative stress status inside the cell. OM cells were seeded on a 96-well plate with a density of 10,000 cells per well three days before the exposure to UFPs for 24-h, and 72-h. At the time of the assay, cell culture media was removed and replaced with 0,5 mM H_2_DCFDA in Hank's Balanced Salt Solution (HBSS) (Gibco #14175-053). As a positive control for ROS formation, right before the assay cells were incubated for 1 h with 500 μM H_2_O_2_ (hydrogen peroxide) in DMEM/F-12 (Gibco #11320-033). Fluorescence was measured with a Clariostar plate reader (BMG Labtech) with excitation at 485 nm and emission at 530 nm at three different timepoints: 0 min, 30 min, and 60 min. Between measurements, the plate was incubated at 37 °C protected from light. After measurements with DCF, to assess the viability, propidium iodide (PI) (Sigma #P4170-25 MG) was added to all wells with a concentration of 1 mg/ml in PBS and incubated for 20 min at 37 °C. ‘First read’ absorbances were measured with excitation 540 nm and emission 610 nm ‘Last read’ was measured after lysing cells with 10 % (v/v) Triton X-100 followed by 20 min incubation at room temperature. Viability was calculated as the difference between last/first values, and this value was used to normalize fluorescence values from 0 min, 30 min, and 60 min. Therefore, the area under the curve (AUC) was calculated solely from living cells. Results are presented as a percentage of positive control.

### 2.8 ATP-assay

ATP was quantified by using ATPLite Luminescence assay (PerkinElmer #6016941). Cells were seeded on a 48-well plate with a density of 40,000 cells per well with four technical replicates one day before the exposure to UFPs. Exposures were done with a concentration of 20 l/ml for 24-h and 72-h. The assay was performed according to the manufacturer's instructions. UFP-containing media was collected and replaced with DMEM/F-12 (Gibco #11320-033), cells were lysed by shaking in an orbital shaker for 10 min at 100–200 rpm and lysed samples were transferred to an opaque 96-well plate (^Optiplate)^ followed by adding the substrate solution and repeating shaking for 5 min. After dark adapting the plate for 10 min, luminescence was measured with a Wallac Victor 1420 plate reader (PerkinElmer, Waltham, USA). A standard curve was generated for each assay. Results are presented as a mean from four technical replicates/conditions/cell lines.

### 2.9 Cell redox-profiling/NAD+/NAHD/NADPH/GSH/GSSG-assay

Quantitative analysis of NAD+, NADH, NADP+, NADPH, and reduced and oxidized glutathione in cultured cells was done as a service in NADMED laboratory (Helsinki, Finland) using a proprietary technology (for further information see www.nadmed.com). Cultured cells were washed on the plate with PBS buffer to remove protein from the culture media followed by the addition of pre-warmed lysis solution to quench cellular metabolism. Lysis solution is a water-based complex mixture of organic solvents, which force protein unfolding at temperatures above +45 °C and release of all non-covalently bound metabolites into solution. Lysed cells were carefully scraped from the plate and the obtained homogenate was transferred to a clean microtube and placed on ice to force protein precipitation. Next, homogenates were frozen at −80 °C and shipped on dry ice to the NADMED laboratory. Before analysis, the homogenates were equilibrated to room temperature and centrifuged at 20000 g for 10 min at +4 °C to remove the protein pellet. Next, NAD+, NADH, NADP+, NADPH, GSH pool, and GSSG were measured individually from every cell extract using modified cyclic enzymatic reactions with colorimetric detection. For normalization of the results, protein content in the sample was measured using the pellet obtained after centrifugation of the homogenate.

### 2.10 Statistical analyses

Statistical analyses were performed with GraphPad Prism 8.0.2 (GraphPad Software Inc. San Diego, CA, USA) software. Statistical outliers were removed based on Grubb's test, p < 0.05. Unpaired Student's t-test assessed statistical differences between vehicle and exposure. In experiments that contained more than two exposures, each exposure (Euro6, A0, A20) was compared to the vehicle by one-way analysis of variance (ANOVA) with Dunnett's multiple comparison test. A two-way analysis of variance (ANOVA) was used to examine the effect of exposure, the difference between health status, and the interaction effect of exposure and health status. All data are presented as means with the standard deviation (SD). An adjusted p-value <0.05 was considered statistically significant. Biorender and the open-source vector graphics editor Inkscape 1.2.1. were used to create graphical illustrations.

## 3 Results

### 3.1 Cellular viability is reduced by UFPs in human OM cells and depends on the source of pollutant and exposure time

**Fig. 1:**
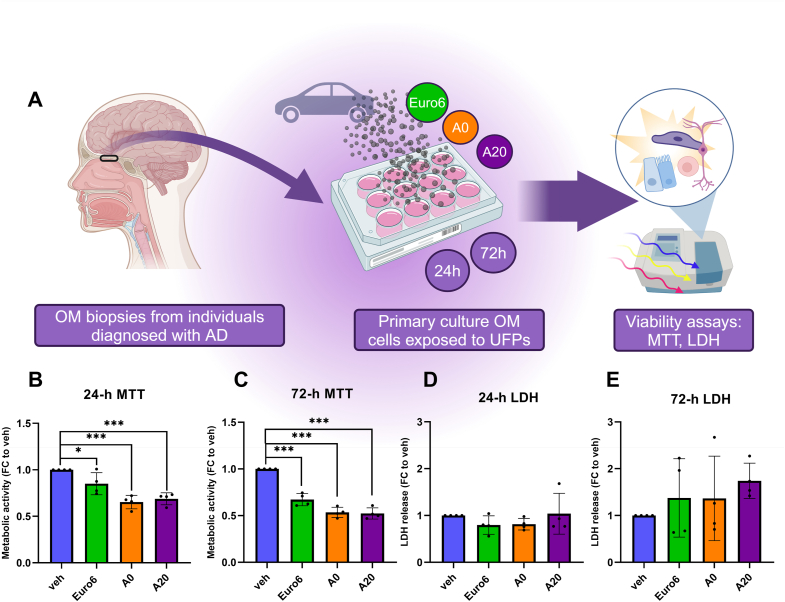
Assessment of cellular viability. (A) Illustration of experimental workflow from receiving the OM biopsy, culturing and exposing cells to UFPs, and assessing cellular viability by MTT and LDH assays. (B, C) MTT reduction assay. OM cells from n = 4 individuals with diagnosed AD were exposed to different UFPs with a concentration of 20 l/ml. Results are presented as a fold change (FC) to vehicle-treated cells, with each cell line compared to its corresponding vehicle. (D, E) LDH release assay from collected cell culture medium after 24-h and 72-h exposure to different UFPs at a concentration of 20 l/ml, AD OM cells (n = 4). Results were calculated as a percentage from lysed cell control (TXT) = absolute death, and presented as an FC to vehicle-treated cells, each cell line compared to its corresponding vehicle. One-way ANOVA, Dunnett's multiple comparisons test * = p < 0.05; *** = p < 0.001.

As described in our previous studies with OM cells derived from cognitively healthy controls, we first defined the lowest effective UFP dose at which a cellular response occurs [36]. We exposed OM cells from different individuals diagnosed with AD (n = 4) to A0, A20, and Euro6 samples (Fig. 1A) at the same concentration of 20 l/ml and performed the MTT reduction assay at the 24-h and 72-h timepoints (Fig. 1B and C; Supplementary Fig. 2). At the 24-h timepoint we observed a statistically significant decrease in metabolic activity compared to the vehicle-treated cells with all UFPs, with A0 and A20 causing stronger responses than Euro6 (35 % [A0; p < 0.001], 31 % [A20; p < 0.001], and 15 % [Euro6; p < 0.05] reductions; Fig. 1B). At the 72-h timepoint, again all exposures resulted in a significant decrease in metabolic activity, with A0 and A20 causing stronger responses than Euro6 (47 %, 48 %, and 33 % reductions, respectively; p < 0.001; Fig. 1C). It is notable that at both timepoints, A0 and A20 resulted in nearly equal decreases in metabolic activity, whereas Euro6 had a reduced effect. Overall, with all UFP exposures, the reduction in cell viability was more prominent at the 72-h timepoint. To determine whether UFPs cause cell death in the AD OM cells, cellular toxicity upon 24-h and 72-h pollutant exposure was assessed by the lactate dehydrogenase (LDH) release assay (Fig. 1D and E). LDH assay was performed from collected cell culture medium from the same OM cell-lines exposed to a 20 l/ml concentration of A0, A20, and Euro6. Lysed cells (Triton X-100 treated) serve as an indicator for absolute cell death (100 %). No significant cytotoxicity was observed in response to any exposure compared to the vehicle-treated cells at either timepoint (Fig. 1D and E). Despite the slight elevation in LDH release at the 72-h timepoint (Fig. 1E), the magnitude of the cellular toxicity observed upon UFP exposures overall was relatively modest. However, individual differences seemed to increase with the longer 72-h exposure, especially with cells derived from one individual being more sensitive to all exposures than others. Taken together, these findings indicate that exposure to all UFPs affects the metabolic activity (as assessed by MTT assay) in OM cells with some time-dependent effects and that exposures to A0 and A20 UFPs elicit the strongest effects, although without causing eminent cellular toxicity. These results correspond to changes observed with cells derived from cognitively healthy controls in our previous study, where A0 and A20 caused a significant decrease in metabolic activity in both timepoints without causing cellular toxicity [36].

### 3.2 RNA-seq reveals a strong mitochondrial response upon UFP exposure

After verifying a suitable concentration to elicit an effect without cytotoxicity, we performed total RNA-Seq on OM cells from different individuals with confirmed diagnoses of AD (n = 6). Sequencing was done at two different timepoints as reported in our previous work with cells derived from cognitively healthy individuals, but in this paper, we focus on the 24-h timepoint, also combining some findings from the previous work [36].

**Fig. 2:**
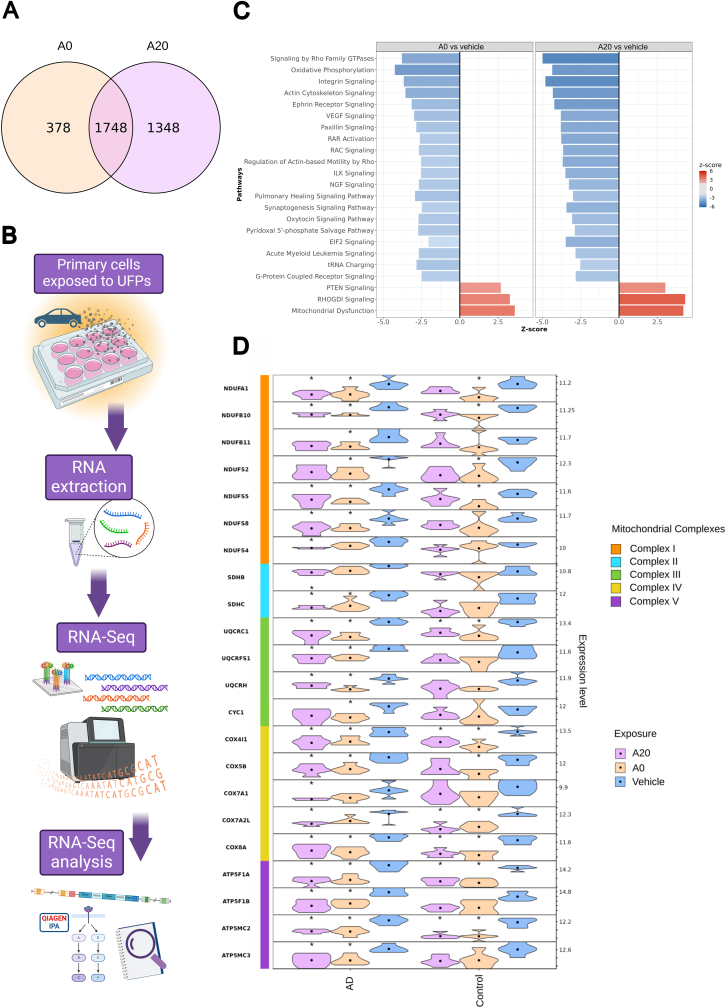
Transcriptomic assessment of 24-h UFP-exposed AD OM cells. (A) Venn diagram presents the overlap and individual DEGs in OM cells exposed to A0 and A20. (B) Illustration of the workflow from exposing OM cells to traffic-derived UFPs, extracting RNA, generation of cDNA-libraries, and performing RNA-Seq and data analysis. (C) Barplot showing the z-scores of the most perturbed Ingenuity Pathways enriched upon exposure to A0 and A20. (D) Stacked violin plot presenting the expression values distribution of a subset of DEGs from OXPHOS-pathway in AD and cognitively healthy control samples exposed to A0, A20, and vehicle. In each violin plot the black dot represents the mean expression value and the asterisk indicates significant (p < 0.05) difference compared to the vehicle.

In total, differential expression analysis at the 24-h timepoint revealed 2126 differentially expressed genes (DEGs) in A0 exposed cells compared to vehicle-treated cells (956 upregulated and 1170 downregulated), while exposure to A20 induced 3096 DEGs (1478 upregulated and 1618 downregulated). There were no genes found to be differentially expressed in Euro6-exposed cells (see Supplementary Table 2 for a full listing of all DEGs). Only DEGs with an adjusted p-value lower than 0.05 were included in the analyses. Fig. 2A presents the overlap of DEGs between A0 and A20, demonstrating shared features of these exposures, although A20 has a notable amount of additional, distinct genes that were not altered by treatment with A0 UFPs.

At the 72-h timepoint, the corresponding results were: 6414 DEGs with A0, 6819 DEGs with A20, and 436 DEGs with Euro6 (see Supplementary Table 2 for a full listing of all DEGs). Collectively, A0 and A20 elicited similar effects in this longer exposure compared to the shorter 24-h exposure, although the magnitude of the responses was increased in the 72-h exposure. Although Euro6 exposure resulted in some DEGs at the 72-h timepoint, it was minor compared to the alterations observed with A0 and A20.

Given the vast amount of DEGs observed with exposures to A0 and A20, we utilized features of Ingenuity Pathway Analysis (IPA) to assess the biological processes perturbed in AD cells exposed to UFPs. Since the majority of DEGs were shared, as shown in the Venn diagram (Fig. 2A) it was expected that most of the pathways affected by exposure would be similar. An illustration of the workflow is presented in Fig. 2B Fig. 2C presents the top 23 (20 inhibited and 3 activated) most altered pathways of interest in AD cells at the 24-h timepoint with exposures to A0 and A20. Pathways were considered significant only with z-scores ≥2 or ≤ −2 combined with -log(p-value) ≥ 1.3. (All pathways from both timepoints are listed in Supplementary Table 3). With both A0 and A20 exposure, most pathways were inhibited and only a few were activated. The most striking effect was found for pathways related to mitochondrial functions, where mitochondrial dysfunction was strongly activated while oxidative phosphorylation (OXPHOS) was inhibited (Fig. 2C). Another significant observation was the activated RHOGDI-signaling and inhibited Signaling by Rho Family GTPases. Other significantly inhibited pathways were Integrin Signaling, Actin Cytoskeleton Signaling, Ephrin Receptor Signaling, VEGF Signaling, Paxillin Signaling, Regulation of Actin-based Motility by Rho, RAC Signaling, ILK Signaling, EIF2 Signaling, and G-Protein Coupled Receptor Signaling. Overall, the magnitude of the responses was increased upon exposure to A20 compared to A0, which corresponded to the number of DEGs found.

At the 72-h timepoint, the IPA enrichment results resembled the 24-h exposures - OXPHOS was the most inhibited and mitochondrial dysfunction the most activated pathway with both exposures, although the responses appeared stronger compared to the shorter timepoint (Supplementary Table 3). The 72-h exposure to Euro6 also resulted in some significant pathways that were similar to those observed with A0 and A20, such as inhibited Actin Cytoskeleton Signaling, Signaling by Rho Family GTPases, and activated RHOGDI-signaling (Supplementary Table 3).

Since based on the RNAseq results the UFPs A0 and A20 seemed to profoundly target mitochondria, we wanted to explore more closely the expression of these pathway-specific genes in OXPHOS (Fig. 2D). We were also interested in comparing these findings to our previous study with cognitively healthy controls, since at the 24-h exposure to A0 we also observed OXPHOS to be among the top 20 inhibited pathways.

At the 24-h timepoint we observed several genes to be downregulated in the various mitochondrial complexes; Complex I (NADH: ubiquinone oxidoreductase): *NDUFA1, NDUFB10, NDUFB11, NDUFS2, NDUFS4, NDUFS8;* Complex II (succinate dehydrogenase): *SDHB, SDHC;* Complex III (coQ-cytochrome c reductase): *UQCRC1, CYC1, UQCRFS1, UQCRH*; Complex IV (COX: cytochrome *c* oxidase): *COX4I1, COX5B, COX7A1, COX7A2L, COX8A;* and Complex V (F_1_F_0_ ATP synthase): *ATP5F1A, ATP5F1B, ATP5MC2, ATP5MC3* (Fig. 2D; see Supplementary Table 4 for listing all found DEGs in OXPHOS pathway and their *log2FC* values). The asterisks in Fig. 2D indicate a significant difference (p < 0.05) between treatment and vehicle. In response to UFPs A0 and A20, we found more altered genes in the OXPHOS pathway in the AD group (18 [A0] and 19 [A20] DEGs) compared to the cognitively healthy control group (11 [A0] and 7 [A20] DEGs; Fig. 2D; Supplementary Table 4). Although individual genes were not so highly downregulated, with *log2FC* values varying from *log2FC -0.19* down to *log2FC -0.53*, having multiple altered genes in the pathway increases the biological significance. While all other genes in the OXPHOS pathway were downregulated in response to A0 and A20 exposures, gene *ENOX1* was found to be upregulated in both cognitively healthy controls (*log2FC 0.90* [A0] and *0.97* [A20]) and ADs (*log2FC 0.82* [A0] and *0.94* [A20]; Fig. 2D; Supplementary Table 4). Based on the deviations in the violin plot it seems that ADs form a more homogenous group in their responses compared to cognitively healthy controls where variation between individuals seems more profound (Fig. 2D). Overall, gene-level alterations in the OXPHOS pathway imply that disturbances are more distinct in the AD group.

Mitochondrial dysfunction genes are presented in a heatmap in Supplementary Fig. 3. IPA listing of mitochondrial dysfunction included overlapping genes with OXPHOS, which is obvious since impaired OXPHOS is one feature of dysfunctional mitochondria, but for clarity, they were removed from the heatmap. Among the most dysregulated genes were e.g. uncoupling protein *UCP2* and glutathione metabolism-related *GSR* and *GSTA1.* In addition to glutathione, other downregulated genes involved in maintaining redox balance were *SOD1* and *SOD2* [44].

At the 72-h timepoint similar, although more severe and vast changes in DEGs were observed in OXPHOS and mitochondrial dysfunction pathways (Supplementary Table 3).

Taken together, these results suggest that exposures to A0 and A20 UFPs affect mitochondrial respiration in OM cells by altering several genes involved in OXPHOS. In addition, the mitochondrial dysfunction pathway revealed several other genes indicating possible mechanisms of how mitochondrial functions are hampered e.g. by altering the redox balance. We proceeded with the 24-h timepoint for more detailed functional analyses.

### 3.3 Exposures to A0 and A20 induce severe disruptions in mitochondrial respiration leading to decreased ATP production

**Fig. 3:**
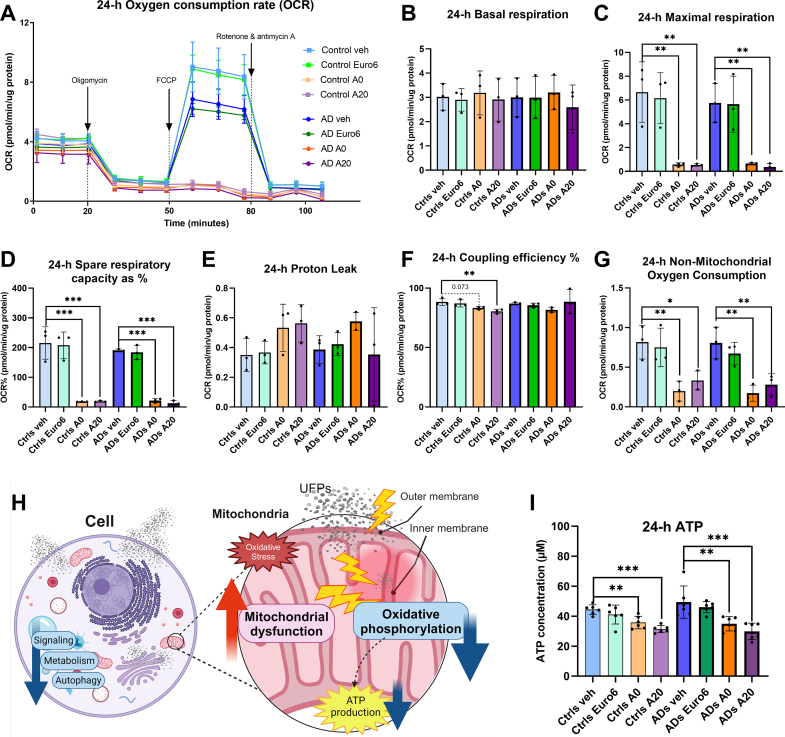
**UFPs reach mitochondria and impair OXPHOS.** (A)–(G) Seahorse Mitostress Test assay to assess mitochondrial functions in OM cells from cognitively healthy controls and ADs exposed to vehicle, Euro6, A0, and A20 for 24-h with a concentration of 20 l/ml and quantified in pmol/minute/μg protein. (A) Line plot with one cognitively healthy control and one AD OM cell line displaying oxygen consumption rate (OCR) at baseline followed by the administration of an ATP synthase inhibitor oligomycin at 20 min, the uncoupler FCCP at 50 min, and a mix of complex I and complex III inhibitors (rotenone and antimycin A at 80 min. All following respiratory parameters (B)–(G) present OM cells from cognitively healthy controls and ADs (n = 3/group) (B) Basal respiration: the last OCR measurement before injection of oligomycin-non-mitochondrial oxygen consumption (C) Maximal Respiration: the maximum OCR that can be achieved after the addition of FCCP (D) Proton Leak: OCR after the addition of oligomycin, indicating remaining basal respiration not coupled to ATP production (E) Spare respiratory capacity as %: maximal OCR – baseline OCR * 100 (F) Coupling Efficiency%: (baseline OCR - OCR after the addition of oligomycin)/baseline OCR * 100 %. (G) Non-mitochondrial oxygen consumption: Minimum rate of OCR after adding rotenone & antimycin A. All exposures are compared to the vehicle-treated cells inside the health status group with One-way ANOVA, Dunnett's multiple comparisons test * = p < 0.05; ** = p < 0.01; *** = p < 0.001. (H) Illustration of how UFPs reach even the mitochondrion and cause multiple effects. (I) ATP concentrations in OM cells after 24-h exposure to the vehicle, Euro6, A0, and A20, presented as μM. Each dot represents cell line n = 6 controls/ADs, calculated as mean from 4 technical replicates. One way ANOVA, Dunnett's multiple comparisons test ** = p < 0.01; *** = p < 0.001.

Since transcriptomic analysis revealed a strong inhibition of the OXPHOS- pathway in exposures to A0 and A20, we assessed the effect of UFPs on mitochondrial respiration by utilizing Seahorse XF technology for analysis of cellular respiration. We exposed OM cells from both cognitively healthy controls and ADs (n = 3/group) to UFPs for 24-h with a concentration of 20 l/ml, similarly as in previous functional assays and RNA-Seq. Fig. 3A presents the oxygen consumption rate (OCR) -curve with one representative line from both groups exposed to vehicle Euro6, A0, and A20. where We did not observe any significant changes in the basal respiration level (level before administration of oligomycin) within the first 20 min of the experiment (Fig. 3B). This supports our earlier conclusions that cells are alive and can function on some level despite the presence of UFPs. Exposure to vehicle or Euro6 emissions did not appear to affect mitochondrial parameters as much as A0 and A20, both in the cognitively healthy control and AD cells.

However, after the addition of uncoupler FCCP at 50 min, when the mitochondrial function should burst and indicate maximal respiratory capacity, we observed a robust response with the vehicle and Euro6 treated cells, but a complete lack of response with A0 and A20 treatment (Fig. 3A). From the bar plot is seen a significant response in maximal respiration in both health status groups exposed to A0 and A20 emissions compared to vehicle-treated cells. The decrease was 92 % [A0 and A20] in cognitively healthy control cells and in ADs: 89 % [A0] and 94 % [A20] (p < 0.01; Fig. 3C). Exposure to Euro6 does not cause significant alterations in maximal respiration compared to vehicle-treated cells in cognitively healthy controls or ADs, but there is notable variation between individual cell lines (Fig. 3C).

As expected, based on the maximal respiration, exposures to A0 and A20 caused a drastic drop in spare respiratory capacity in both health status groups (in controls: 92 % [A0] and 91 % [A20], and in ADs: 87 % [A0] and 93 % [A20], p < 0.001), while the response to Euro6 did not deviate significantly from the vehicle-treated cells in either of the groups (Fig. 3D). Proton leak was found to be slightly elevated in the cognitively healthy controls with exposures to A0 and A20, and in ADs exposed to A0 compared to vehicle, but results were not statistically significant (Fig. 3E). Exposure to Euro6 did not elicit a significant change in either group.

Coupling efficiency (Fig. 3F) shows a slight but significant decrease compared to the vehicle-treated cells in response to A20 in the cognitively healthy controls (9 % p < 0.001), however response to A0 did not quite reach the statistical significance (p < 0.073). In the ADs exposure to A0 or A20 did not lead to significant decrease of coupling efficiency. Again, no notable effect was seen with exposure to Euro6 emissions compared to vehicle-treated cells in either health status group.

Non-mitochondrial oxygen consumption was strongly affected by A0 and A20 compared to the vehicle-treated cells in both groups, being slightly more apparent in ADs (Fig. 3G). Compared to the vehicle-treated cells, observed decreases were in cognitively healthy controls 75 % (p < 0.01) with A0, 59 % with A20 (p < 0.05), and in ADs 79 % with A0 and 65 % with A20 (p < 0.01). The illustrative Fig. 3H presents how UFPs penetrate a cell, and due to their small size, reach even the mitochondrial membranes, as has been previously validated with lung macrophages [45]. Lastly, ATP, the end product of OXPHOS, was quantified by using an ATPLite Luminescence assay. At the 24-h timepoint exposures to A0 and A20 resulted in a significant decrease in ATP production compared to vehicle-treated cells both in cognitively healthy controls (19 % [A0] p < 0.01; and 29 % [A20] p < 0.001; Fig. 3I) and ADs (29 % [A0] p < 0.01; and 39 % [A20], p < 0.001; Fig. 3I). Euro6 did not elicit a notable decrease in ATP production in either of the health status groups.

Taken together, these results indicate that in response to A0 and A20 exposures mitochondrial respiration is heavily affected. Exposures to A0 and A20 decrease maximal respiration, spare respiratory capacity, coupling efficiency, non-mitochondrial oxygen consumption, and ATP production compared to vehicle-treated cells. Euro6 does not elicit any notable differences in respiratory parameters compared to the vehicle-treated cells.

### 3.4 Exposure to UFPs disturbs redox balance leading to oxidative stress in OM cells

**Fig. 4:**
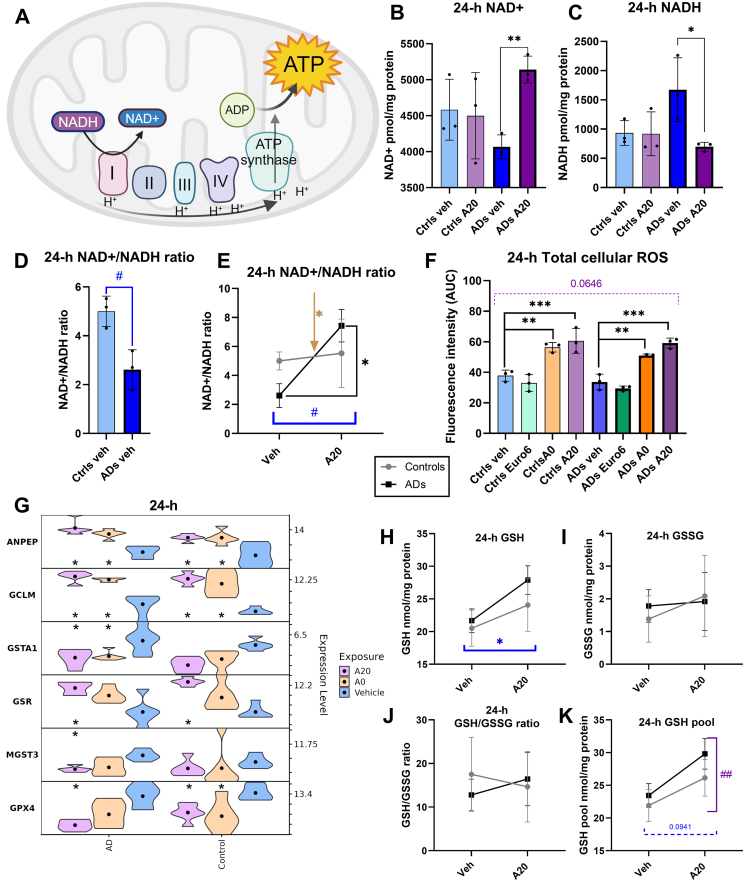
(A) Illustration of the ETC with NADH-molecules as electron donors and releasing protons into the intermembrane space, finally resulting in pumping them back to the mitochondrial matrix while generating ATP (B) Amount of oxidized form (NAD+) and (C) reduced form (NADH) of nicotinamide adenine dinucleotide in cognitively healthy control/AD cells exposed to vehicle and A20 presented as pmol/mg of protein, Student's t-test, * = p < 0.05; ** = p < 0.01. (D) NAD+/NADH-ratio in cognitively healthy control/AD cells exposed to vehicle, Student's t-test # = p < 0.05. (E) NAD+/NADH-ratio presented as a grouped line plot illustrating the statistically significant effect of the exposure (# = p < 0.05), and the interaction effect of exposure and health status (* = p < 0.05) two-way ANOVA. In addition, there is a significant difference between AD veh – AD A20 (p < 0.05), two-way ANOVA followed by Sidak's multiple comparison test. (F) Total cellular ROS measured from the OM cells from cognitively healthy controls and ADs (n = 3/group) exposed to veh, Euro6, A0, and A20 for 24-h with a concentration of 20 l/ml. Results were calculated as AUC from fluorescence intensity and presented as a percentage from positive cell control (500 μM H_2_O_2_ – treated) for absolute ROS formation. Asterisks indicate the difference compared to vehicle. One-way ANOVA, Dunnett's multiple comparisons test ** = p < 0.01; *** = p < 0.001. The group effect assessed with two-way ANOVA did not quite reach statistical significance (p < 0.0646). (G) Violin plot presenting DEGs found in the transcriptomic analysis involved in glutathione metabolism, an asterisk indicates a significant (p < 0.05) difference compared to vehicle. (H) GSH in cognitively healthy controls and ADs exposed to vehicle and A20, presented as nmol/mg protein. Two-way ANOVA, significant exposure effect * = p < 0.05. (I) GSSG in cognitively healthy control and AD cells exposed to vehicle and A20, presented as nmol/mg protein. (J) GSH/GSSG ratio of cognitively healthy control and AD cells exposed to vehicle and A20. (K) GSH pool in cognitively healthy controls and ADs exposed to vehicle and A20, presented as nmol/mg protein. Significant health status effect, but exposure effect did not reach statistical significance (p < 0.0941), two-way ANOVA ## = p < 0.01.

We next examined whether the changes observed in transcriptomic analyses and functional mitochondrial measurements manifest in alterations to the cell redox profile and key metabolites. Fig. 4A presents a graphical illustration of the ETC with NADH-molecules as electron donors and releasing protons into the intermembrane space, finally resulting in ATP synthase pumping them back to the mitochondrial matrix and generating ATP [46].

Since the most prominent effects on the OXPHOS were observed with exposure to A20, we were interested in exploring in more detail how it affects nicotinamide adenine dinucleotide (NAD) metabolism and the ratio of oxidized (NAD+) and reduced (NADH) forms, compared to vehicle-treated cells. We observed a significant increase (26 %, p < 0.01) in the NAD + level compared to vehicle in AD cells exposed to A20 at 20 l/ml for 24-h, but not in cognitively healthy control cells (Fig. 4B). Correspondingly, the level of NADH was significantly decreased (58 %, p < 0.05) compared to vehicle in AD cells upon exposure to A20 (Fig. 4C). In cognitively healthy controlsNADH remained unaltered (Fig. 4C). Interestingly, a closer examination of the NAD+/NADH ratio comparing only vehicle-treated cognitively healthy controls and AD cells revealed a statistically significant difference between the two health status groups already at baseline, without UFP exposure (48 %, p < 0.05; Fig. 4D). As expected, we did not observe notable differences in cognitively healthy controls in the NAD+/NADH-ratio between exposures (Fig. 4E). Instead, in ADs, a significant difference in the NAD+/NADH-ratio between exposures to the vehicle and A20 was seen (185 % increase, p < 0.05; Fig. 4E). Notable is also the interaction effect between exposure and health status, presented in the line plot as lines that cross each other (Fig. 4E). This interaction effect indicates that the exposure affects the two health status groups in opposite directions, further highlighting the difference in responses between cells derived from cognitively healthy and individuals with AD. Overall, it is evident that in AD cells exposure to UFPs causes eminent effects on the NAD balance.

Given the UFP-induced alterations to mitochondrial function and metabolites, we next evaluated the total ROS levels and oxidative stress status in UFP-exposed OM-cells. In both health status groups, we saw a significant increase in ROS levels detected by H2DCFDA dye in response to A0 (in controls 49 % and ADs 51 %, p < 0.01) and A20 (in controls 60 % and ADs 76 %, p < 0.001) exposure, while Euro6 did not elicit changes (Fig. 4F). When comparing health status groups together, we observed a slight but not quite significant (p < 0.0646) difference between cognitively healthy controls and ADs.

Glutathione is an important cellular antioxidant defense against reactive oxygen species, thereby we measured both the reduced form of glutathione (GSH) and oxidized form (GSSG) in the UFP-exposed cells. Transcriptomic data revealed several dysregulated genes involved in GSH metabolism in response to A0 and A20 exposures, such as *ANPEP, GCLM, GSTA1, GSR, GPX4, and MGST3* (Fig. 4G). We observed an increase in the GSH upon exposure to A20 compared to vehicle-treated cells, with a more pronounced difference in AD cells (in controls 17 % and ADs 29 %; Fig. 4H).

In the level of the GSSG (Fig. 4I), or in the GHS/GSSG ratio (Fig. 4J) were not observed any statistically significant alterations in response to exposure or between the health status groups. However, the GSH pool (Fig. 4K) revealed a significant health status effect (p < 0.01). This suggests that compared to vehicle-treated cells, exposure to A20 has some effect on the glutathione metabolism in OM cells and that our study groups, cognitively healthy controls, and ADs differ from each other.

Another interest of ours was to determine the concentration of nicotinamide adenine dinucleotide phosphate (NADP) to understand better its role in the redox balance of OM cells exposed to UFPs. However, slight but not statistically significant changes were observed in response to the exposure or between the health status groups. This data is presented in Supplementary Fig. 4.

Taken together, the UFP-induced alterations observed in NAD, NADP, and GSH metabolism, coupled with elevated total ROS provide strong evidence that UFPs disturb the redox balance by several mechanisms. Furthermore, some of the responses differ slightly between OM cells derived from cognitively healthy controls and AD individuals.

## 4 Discussion

The aim of this study was to investigate alterations in the functions of mitochondria caused by traffic-derived UFPs and their possible connection to AD by utilizing a human *in vitro* model of OM. We exposed OM cells from individuals diagnosed with AD to UFPs and coupled transcriptomic findings with functional assessment together with the data from our previous work using OM cells derived from cognitively healthy donors [36]. As reported previously, all the pollutant samples were UFPs with the majority of particles being ≤0.1 μm by aerodynamic diameter. A0 and A20 had larger amounts of the smallest particles, as well as high levels of nitrogen species and a wide repertoire of different PAHs compared to the Euro6. The Euro6 sample was found to be very clean, containing only incremental numbers of nitrogen species [36].

In the current study, we report that several mitochondrial processes are perturbed both in cells derived from cognitively healthy controls and in individuals diagnosed with AD and that different aftertreatment devices of the engines have a major impact on the outcomes. First, the metabolic activity of AD cells was altered upon UFP exposure in a time-dependent manner, and most so with A0 and A20, but still without overt cellular toxicity. Notable was the response of AD cells to Euro6, which was not observed with cognitively healthy controls as reported [36].

Secondly, transcriptomic analysis revealed a strong response in AD OM cells exposed to UFPs A0 and A20, but not to Euro6. Although the results of the current study were similar to the observations of our previous study with cognitively healthy control cells [36], the amount of DEGs induced by A0 and A20 in AD cells exceeded the amounts found in control cells. By utilizing Ingenuity Pathway Analysis, we discovered that the most significant inhibited pathway with these exposures was OXPHOS, and the most activated pathway was mitochondrial dysfunction. Since similar features were also observed within the cognitively healthy controls, we decided to further explore these changes by including both study groups at the 24-h timepoint. By utilizing RNA-Seq, transcriptomic alterations on mitochondrial functions induced by air pollutants have only been investigated by a few, however, the findings of these studies are similar to ours, indicating the important role of mitochondria in air pollutant-mediated disturbances in cells [[47], [48], [49]]. Although several mitochondrial disturbances leading to AD have been described [20] we decided to mainly focus on the main duty of mitochondria, the production of ATP via OXPHOS [50]. Our data revealed that several genes from all OXPHOS complexes I–V were inhibited upon exposure. PM exposure has been previously shown to decrease mitochondrial respiration by inhibiting complex II in macrophages [51].

As the main function of the mitochondria is to transform the energy stored in biomolecules by carefully orchestrated reactions to generate ATP, our main interest lies in the inner mitochondrial membrane (IMM) where OXPHOS takes place, comprising of electron transfer chain (ETC) and chemiosmosis to generate ATP from ADP [52]. Mitochondrial respiration assessed by Seahorse XF Cell Mito Stress Test was heavily affected by the exposure to A0 and A20 UFPs. In the basal respiration level, we did not observe clear differences between the used UFPs, indicating that OM cells can maintain some basic functions despite the particles. A decrease in basal respiration has previously been reported with OM cells exposed to urban PM2.5 and PM10 particles [24], in human neuroblastoma (SH-SY5Y) cells exposed to benzo(*a*)pyrene [53] and in SH-SY5Y cells and *ex vivo* in hepatic explants from mice exposed to traffic-related UFPs [18]. However, in the study done with OM cells [24], exposure to the smallest fraction of PM1 did not notably affect the basal respiration level, which might indicate together with the findings from the current study that changes observed upon exposure to the smallest fractions, PM1, and UFPs are more subtle. However, while we did not see an increase in OCR levels in the cells exposed to A0 and A20, maximal respiration and spare respiratory capacity were highly reduced. Benzo(*a*)pyrene has also been shown to impair maximal respiration and spare respiratory capacity in SH-SY5Y cells with time and dose-dependent matter [53]. The spare respiratory capacity indicates a cell's fitness and capacity to meet sudden changes in energy demands in response to stress reactions [52]. As also observed in the transcriptomic data, several complex IV genes were inhibited in response to A0 and A20. The mammalian complex IV consists of 14 subunits, and it is well documented that problems in any of these subunits can lead to serious consequences for the complex activity [54]. It appears that the stress caused by A0 and A20 UFPs impairs OM cells' ability to meet the requirements of the sudden need for energy, possibly by disturbing OXPHOS complex IV. It has been shown that the environmental toxicant TCDD causes the dysregulation of genes involved in the ETC, possibly in an aryl hydrocarbon receptor (AHR)-dependent manner [55]. As previously reported, A0 and A20 samples contained high amounts of different PAHs [36], known substrates of AHR [56] suggesting that disturbances in ETC of OM could be mediated via AHR. We also observed a strong response to A0 and A20 in non-mitochondrial oxygen consumption, and this effect was slightly more profound in AD cells. This finding indicates that A0 and A20 can potentially impair also other routes besides OXPHOS to produce ATP.

Proton leak was found to be slightly increased in response to A0 and A20 compared to vehicle-exposed cells in cognitively healthy controls and in ADs exposed to A0. Proton leak is a normal phenomenon where some of the protons leak through the mitochondrial membranes to the matrix and one of the mechanisms that makes the coupling of substrate oxygen and ATP generation incomplete [52,57,58]. Increased proton leak is connected to exposure to PAHs such as benzo(*a*)pyrene and anthracene in studies with fish [[59], [60], [61]], further supporting that PAHs may have a role in mitochondrial dysfunction in OM cells. Previously it has been shown that exposure to Residual Oil Fly Ash (ROFA), a PM surrogate rich in transition metals, increased proton leak as well as diminished spare respiratory capacity and coupling efficiency in RAW 264.7 macrophages [62].

In the last step in OXPHOS, in the chemiosmotic phase, the transfer of protons from IMS to the mitochondrial matrix by Complex V (ATP synthase) generates energy to produce ATP [52]. Reductions in ATP levels in response to A0 and A20 exposures were also observed, as assessed by the ATP Luminescence assay. Decreased spare respiratory capacity leading to impaired capability to produce ATP is correlated with several pathologies including neurodegenerative disorders [63], yet here we did not observe significant differences between cognitively healthy controls and AD cells. We also saw alterations in several genes involved in Complex V, which may directly impair ATP generation. It has been reported that sulfur dioxide represses the expression of complex IV and V subunits in rat lungs [21]. Increased proton leak may also affect the decreased production of ATP by reducing coupling, which was correspondingly seen in the reduction in coupling efficiency % in response to A20 exposure in cognitively healthy controls.

Given that NADH serves as a key hydride donor in the ETC to provide ATP [64], an increase in the NAD+/NADH ratio in response to A20 exposure could be one reason for the decreased level of ATP in AD cells. However, there was no significant difference in NAD+/NADH ratio in cognitively healthy controls and as we saw a similar decrease in ATP levels in response to A0 and A20 exposures, the increase in NAD+/NADH ratio cannot fully explain the ATP depletion observed in both health status groups. Overall, it seems that there are only small differences between cognitively healthy control and AD cells in some respiratory parameters in responses to A0 and A20. Collectively, these results suggest that the UFPs A0 and A20 are inducing severe disruption in several respiratory parameters, indicating impaired mitochondrial respiration leading to diminished ATP production.

Examination of the cellular redox profile was conducted by quantifying key metabolites, which revealed alterations in NAD and GSH metabolism in response to A20. We found a significant elevation in NAD+/NADH ratio in A20 exposed AD cells, but not in cognitively healthy control cells. Within the cognitively healthy control group, notable was the donor-to-donor variation in NAD+ and NADH levels upon exposure to A20, highlighting individual differences in the responses to exposure. Interestingly, NAD + levels were observed to be lower in vehicle-exposed AD cells compared to cognitively healthy controls, which highlights the difference already in the experimental baseline. This correlates with existing knowledge that reduced NAD + levels are associated with normal aging and neurodegenerative diseases [65] and impaired NAD + metabolism can result in diseases [64]. Furthermore, NAD + has been shown to play an important role in neuronal protection and inhibiting AD-related pathological changes in animal models [65,66]. Thus, in line with the literature, our results suggest that AD-related alterations in NAD + levels can be seen in OM cells.

Despite its vital role in OXPHOS, NAD+, and its metabolites also have critical functions regulating cells' ability to adapt to environmental insults, such as genotoxic factors, infection, inflammation, and xenobiotics [64]. Given that NAD+ is an important molecule in the production of NADP and further GSH as a protective mechanism to defend against excessive ROS [65], it could be one reason for the elevation observed in AD cells. Upon exposure to A0 and A20, transcriptomic data revealed in both AD cells and cognitively healthy controls an upregulation of *ENOX1*, a gene regulating NAD homeostasis via oxidization of NADH to NAD + [67]. However, this did not correlate with cognitively healthy controls where the NAD+/NADH ratio remained nearly unchanged despite the exposure. An imbalance in the NAD+/NADH ratio has been connected to impaired complex I activity leading to reduced ATP production and maximal respiration in type 2 diabetes patients [68]. However, the impact of air pollutants on the NAD+/NADH ratio is far less studied, and few mice studies report opposite effects where PM exposure decreased NAD + levels [69,70]. It seems that A20 treatment causes an imbalance in NAD metabolism in AD cells but not in cognitively healthy control cells, which may indicate that something in the regulation of NAD+/NADH - homeostasis is impaired in AD cells. It is possible that the strong response of AD cells to A20 exposure is a compensatory reaction to fill the need for NAD+. As we observed the interaction between exposure and health status in NAD+/NADH ratio, it highlights the difference in the response between cognitively healthy controls and ADs.

The majority of ROS originate from the respiratory chain in mitochondria as unwanted side products. ROS is a collective term for all O_2_ -originated species that are more reactive than O_2_. While a more detailed examination of different species was beyond the scope of the current study, we use the term ROS to collectively describe all O_2_ -derivatives, whether they are considered radical species or not. ROS serve as important signaling molecules regulating various cellular functions, and thus there are fine-tuned regulatory systems to maintain this balance in the cells [44,71,72]. We observed an elevation of total ROS level in cells exposed to A0 and A20. As the vast majority of ROS are produced by complex I using NADH as an electron donor [44,64], it is possible that the increased NAD+/NADH-ratio in A20 exposed AD cells have a connection to the elevation of ROS [58]. However, in cognitively healthy controls, we did not see a strong effect in NAD+/NADH-ratio in response to A20 exposure, which does not explain the increased ROS observed in A20 exposed cognitively healthy controls. It is also reported that if complex IV is blocked, preventing electron flow towards O_2_ reduction, electron transfer can be reversed back to complex I and result in an extremely powerful production of superoxide anion (O_2_^•^^−^) [58]. One mechanism protecting from this is a proton leak, which can be induced or suppressed by specific inner membrane proteins such as uncoupling proteins (UCPs) [52,57,58]. In addition to its role in reducing mitochondrial coupling efficiency, the function of UCP2 is largely focused on regulating ROS levels, therefore protecting cells from oxidative damage [52]. We found the gene *UCP2* to be highly downregulated in response to A0 and A20 in both cognitively healthy control and AD cells. Induction of UCP2 with increased ROS levels has previously been shown in rat c6 glioma cells upon exposure to diesel exhaust particles [73]. In mice models the absence of UCP2 has been connected to persistent oxidative stress [57]. UCP2 is expressed in the brain, and its dysfunction is connected to the development of AD. The possible therapeutic potential of UCPs in neurodegenerative diseases has been acknowledged, further highlighting their importance as regulators of ROS production [74]. Since UCP2 is expressed rather ubiquitously, including in the brain, this finding on the OM cells may reflect alterations also in the development of AD in response to air pollution. Our transcriptomic data also revealed the downregulation of superoxide dismutases *SOD1* and *SOD2,* important enzymes metabolizing O_2_^•^−to a less reactive form [44]. *SOD1* was found to be downregulated by A0 in both health status groups and *SOD2* downregulated by A20 in the AD group. As the cell line models also report decreased SOD activity after 24-h exposure to PM2.5 [23,75], in line with the literature, these results indicate that UFPs cause disturbances in ROS balance.

One of the systems maintaining the redox balance is the glutathione system comprising GSH combined with several enzymes [76]. The majority of GSH in normal physiological conditions is in its reduced form, and an increase in GSSG indicates oxidative stress, and consequently the deceased ratio of GSH/GSSG is reported to be associated with AD [76,77] as well as in air pollution studies *in vitro* [62]. In the current study, we observed a significant elevation in the reduced form of GSH in response to A20. Transcriptomic data also revealed dysregulation of several genes involved in GSH metabolism.

Although we did not see a significant effect in the GSH/GSSG ratio, the GSH pool was found to be elevated in response to A20 with both cognitively healthy controls and AD cells, however, this was more prominent in AD cells. As known, oxidative stress induces 10.13039/501100022272GSH synthesis as part of antioxidant defense [44] supported by elevated NAD + levels which further enhance the 10.13039/501100022272GSH synthesis [64]. Thus, alterations in genes regulating GSH metabolism coupled with increased NAD+, GSH, and GSH pool indicate that exposure to A20 UFP triggers GSH-mediated stress response in OM cells. It is possible that in response to A20 UFP exposure, antioxidant defense mechanisms have not yet reacted to relieve the insult or they get overwhelmed and/or disturbed, inducing ROS accumulation and leading to oxidative stress.

Collectively, these results shed light on UFP-induced disturbances to the functions of the mitochondria on human OM cells, by utilizing RNA-Seq corroborated with functional assessment. It is evident that the engine having state-of-the-art aftertreatment devices, which reduce emissions, reduces adverse cellular effects in the OM *in vitro* as seen with two samples containing the same fuel but a different engine: Euro6 vs. unfiltered raw exhaust of A0. In addition, there were changes between A0 and A20 raw exhausts, suggesting that petroleum diesel is inducing more complications to mitochondrial functions in OM cells *in vitro* than renewable diesel. However, this study has some limitations. As reviewed by Murphy and colleagues [72], we acknowledge that OM cells *in vitro* most probably produce more ROS than *in vivo*. Therefore, our measurements of total ROS were indicative of overall oxidative stress in response to UFPs, with the main purpose lying in the comparison of responses between different UFPs with varying particulate and chemical compositions. We also acknowledge that submerged monolayer cell culture is not optimal for air pollution studies, considering the different cellular morphology compared to what can be achieved in air-liquid interface cultures [78]. Also, as the transmission of pollutants to cells is rather unlimited via liquid in submerged cultures, it does not represent fully the air-mediated transmission of particles [79], and can diminish some of the possible features of air pollutants. Also, a rather small human-based sample size and a limited amount of collected and characterized UFPs set limitations to the execution per se. However, even if OM cells from different individuals react to exposures differently, the lack of statistical significance does not mean that there is no biological difference within the individual cell line. We also acknowledge that using the volumetric dosing of emissions leads to completely different masses of UFPs loaded on cells, which might partly explain the observed differences between A0 and A20. However, we consider volumetric dosing important when estimating the total load of emitted particles into the environment due to different fuels/engines. Taken together, the strength of this study lies in the primary cells derived from human nasal biopsies, in which metabolism might still be closer to the cells *in situ* instead of representing the Warburg effect [80]. Furthermore, the same OM cell model derived from cognitively healthy individuals, showed alterations in the genes involved in xenobiotic metabolism, olfactory signaling, and epithelial integrity, indicating the possibility of UFPs mediating harmful effects on the brain through the olfactory route [36]. Using physiologically relevant primary cells gives insights into the mechanisms of how UFPs may impair the OXPHOS and redox balance in OM possibly also reflecting the changes occurring in the CNS in response to air pollutants.

## 5 Conclusion

In summary, we provide molecular-level evidence of mechanisms of how the UFPs, the smallest contributors to air pollution, may hinder the function of the mitochondria of OM cells. The findings of the current study corroborate that especially engine aftertreatment systems, but also fuel quality have a major impact on the adverse effects observed in the mitochondrial functions of OM cells *in vitro*. As the transcriptomic data indicated, the constituents of each emission have clear effects on the response observed also in AD OM cells. Overall, A20 induced the most drastic effects closely followed by A0, while Euro6 exhaust caused only negligible changes in OM cells in both health status groups. We provide evidence that traffic-derived UFPs A20 and A0 can reach even the IMS and impair OXPHOS by disturbing all complexes, resulting in decreased maximal respiration, spare respiratory capacity, coupling efficiency, and non-mitochondrial oxygen consumption, ATP production as well as increased oxidative stress. In addition, activation of mitochondrial dysfunction-pathway coupled with functional quantification implicated disruption of redox balance in response to A0 and A20. Responses observed in AD cells were slightly deviating from the cognitively healthy controls regarding some respiratory parameters as well as NAD and GSH metabolism, suggesting AD-related alterations in OM cells upon exposure to UFPs.

## Availability of data

The RNA sequencing data regarding individuals diagnosed with Alzheimer’s disease presented in this study are available from the European Genome-phenome Archive (EGA, https://ega-archive.org/) on request from the corresponding author. The RNA-sequencing data of cognitively healthy control cells are reused from previously published work by Mussalo and colleagues (2023) [36].

## CRediT authorship contribution statement

**Laura Mussalo:** Writing – review & editing, Writing – original draft, Visualization, Investigation, Funding acquisition, Conceptualization. **Riikka Lampinen:** Writing – review & editing, Investigation, Conceptualization. **Simone Avesani:** Visualization, Resources, Formal analysis. **Táňa Závodná:** Investigation. **Zdeněk Krejčík:** Investigation. **Juho Kalapudas:** Resources. **Elina Penttilä:** Resources. **Heikki Löppönen:** Resources. **Anne M. Koivisto:** Resources. **Tarja Malm:** Resources, Conceptualization. **Jan Topinka:** Resources, Funding acquisition. **Rosalba Giugno:** Resources, Funding acquisition, Formal analysis. **Pasi Jalava:** Writing – review & editing, Supervision, Project administration, Funding acquisition, Conceptualization. **Katja M. Kanninen:** Writing – review & editing, Supervision, Project administration, Methodology, Funding acquisition, Conceptualization.

## Declaration of competing interest

The authors declare that they have no known competing financial interests or personal relationships that could have appeared to influence the work reported in this paper.

## Data Availability

Data will be made available on request.
