# Supplementary material for: Traffic-related ultrafine particles impair mitochondrial functions in human olfactory mucosa cells – Implications for Alzheimer's disease
Source: Redox Biol. 2024 Jul 19;75:103272. doi: 10.1016/j.redox.2024.103272 (PMC11321383; doi:10.1016/j.redox.2024.103272)
Supplement: Multimedia component 1 [file mmc1.docx]

**Supplementary Table 1. UFP volume conversion to mass.**

| **UFP** | **Average volume** | **20 l/ml corresponds to mass** |
| --- | --- | --- |
| A0 | 0.558 m^3^/mg (558 l/mg/tube) | 35,84ug |
| A20 | 0.377 m^3^/mg (377 l/mg/tube) | 53,05ug |
| Euro6 | 0.5 m^3^ (500l/tube) | N/A (PM number too low) |

**A20: To collect 1mg of sample requires 377 l of exhaust run through the filter.**

**A0: To collect 1mg of sample requires 558 l of exhaust run through the filter.**

**Euro6: From the collected 500 l (or even from 1000 l/ tube ) it was impossible to define the mass due to too low amounts of particles in the sample.**

**Supplementary Table 2 (separate Excel file) – A full listing of all DEGs.**

The supplementary Excel file includes all differentially expressed genes (DEGs) found from 24-h exposures to A0 (Sheet 1) and A20 (Sheet 2), and from 72-h exposures to A0 (Sheet 3), A20 (Sheet 4), and Euro6 (Sheet 5).

**Supplementary Table 3 (separate Excel file) – A full listing of Ingenuity Pathway Analysis.**

The supplementary Excel file includes all IPA Pathways found from 24-h exposures to A0 (Sheet 1) and A20 (Sheet 2), and from 72-h exposures to A0 (Sheet 3), A20 (Sheet 4), and Euro6 (Sheet 5).

**Supplementary Table 4.** Listing of all significant (p<0.05) DEGs and their log2FC-values which are listed in the OXPHOS Ingenuity pathway analysis (IPA^®^).

|  |  | controls (log2FC) | | ADs (log2FC) | |
| --- | --- | --- | --- | --- | --- |
|  |  | **A0** | **A20** | **A0** | **A20** |
| Complex I | *NDUFA1* | *-0,425* |  | *-0,331* | *-0,331* |
|  | *NDUFB10* | *-0,393* |  | *-0,323* | *-0,311* |
|  | *NDUFB11* |  |  | *-0,477* |  |
|  | *NDUFS2* | *-0,192* |  | *-0,207* |  |
|  | *NDUFS4* |  |  |  | *-0,445* |
|  | *NDUFS8* |  |  | *-0,413* | *-0,428* |
| Complex II | *SDHB* |  |  |  | *-0,234* |
|  | *SDHC* |  |  | *-0,203* | *-0,245* |
| Complex III | *UQCRC1* | *-0,479* | *-0,377* | *-0,531* | *-0,478* |
|  | *CYC1* |  |  | *-0,418* |  |
|  | *UQCRFS1* |  |  | *-0,300* | *-0,313* |
|  | *UQCRH* |  |  | *-0,333* | *-0,222* |
| Complex IV | *COX4I1* | *-0,508* | *-0,349* | *-0,319* | *-0,363* |
|  | *COX5B* | *-0,324* |  | *-0,274* | *-0,292* |
|  | *COX7A1* |  |  |  | *-0,432* |
|  | *COX7A2L* | *-0,399* | *-0,472* |  | *-0,317* |
|  | *COX8A* | *-0,408* | *-0,373* | *-0,402* | *-0,361* |
| Complex V | *ATP5F1A* | *-0,342* | *-0,306* | *-0,338* | *-0,370* |
|  | *ATP5F1B* |  |  | *-0,242* | *-0,295* |
|  | *ATP5MC2* | *-0,347* | *-0,345* | *-0,279* | *-0,276* |
|  | *ATP5MC3* |  |  | *-0,284* | *-0,281* |
|  | *ENOX1* | *0,902* | *0,969* | *0,817* | *0,940* |

**Supplementary Figure 1. Illustration of the different samples used in the study (data adapted from Mussalo and colleagues, 2023).**


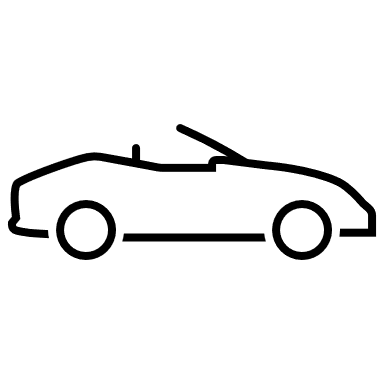

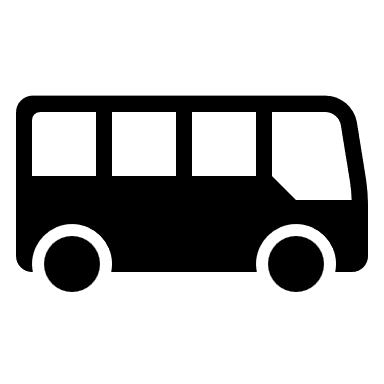

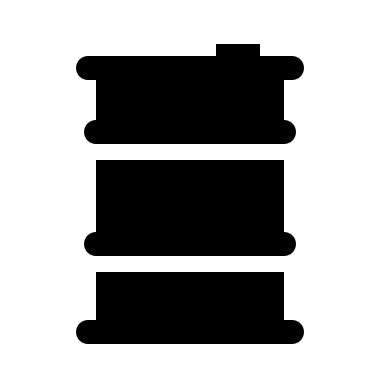

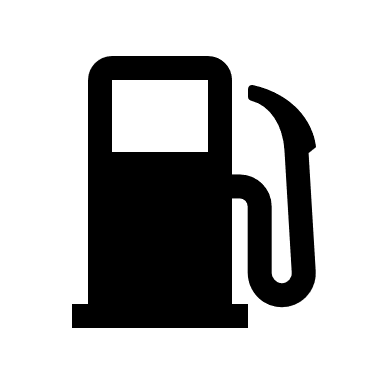

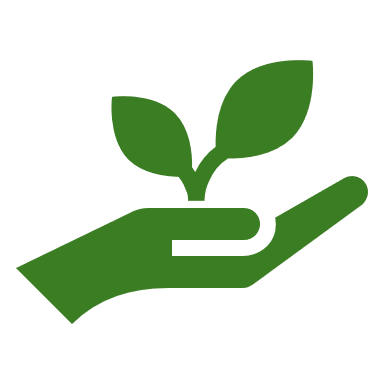

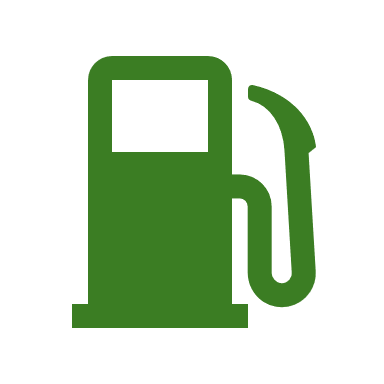


**A20**

Petroleum (fossil) diesel

Renewable diesel

Euro-6-d-temp engine (with diesel particulate filter [DFP] and Selective Catalytic Reduction [SCR])

**Modern Heavy duty engine AGCO 44 AWIC (without any engine aftertreatment devices)**

**A0**

**Euro6**

3 different samples from exhausts of diesel engines: A20 = fossil diesel, run with a heavy-duty diesel engine (HDE) without any exhaust aftertreatment devices. A0 = renewable diesel run with a heavy-duty diesel engine (HDE) without any aftertreatment devices. Euro6 = renewable diesel run with 2019 model diesel passenger car (DI-E6d) equipped with Diesel Particulate Filter (DPF) and a Selective Catalytic Reduction (SCR).

**Supplementary Figure 2. MTT reduction assay for each individual AD cell line in 24-h and 72-h timepoint.**


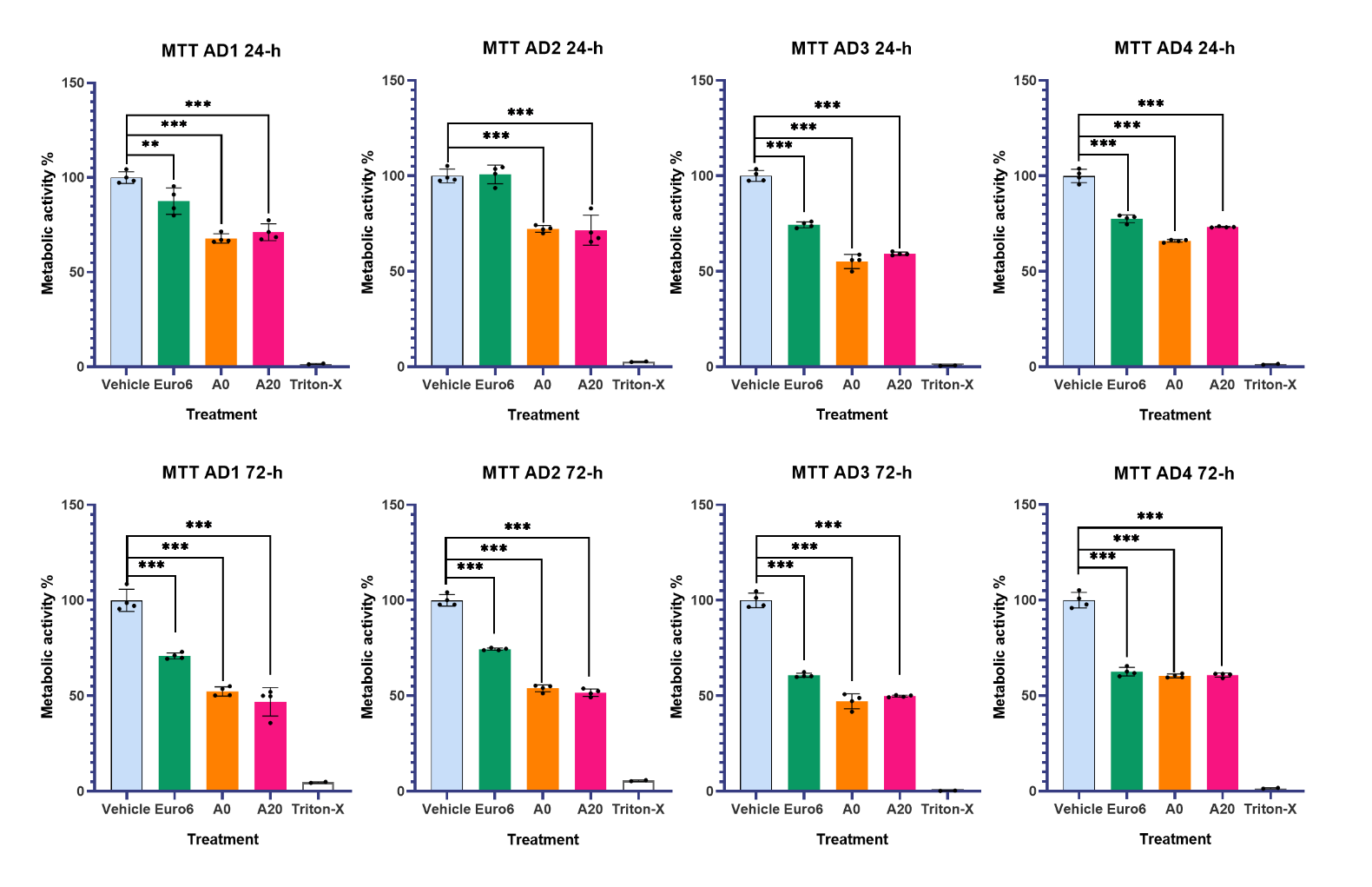


MTT reduction assay done separately for each individual AD OM cell line at 24-h and 72-h timepoints, different individuals named as AD1, AD2, AD3, and AD4. OM cells were exposed to different UFPs (Euro6, A0, and A20) with a concentration of 20 l/ml, and results are normalized to the vehicle-treated cells, which are presented as having 100% metabolic activity. One-way ANOVA, Dunnett’s multiple comparisons test **= p<0.01; ***=p<0.001.

**Supplementary Figure 3. Heatmap of genes involved in mitochondrial dysfunction pathway.**


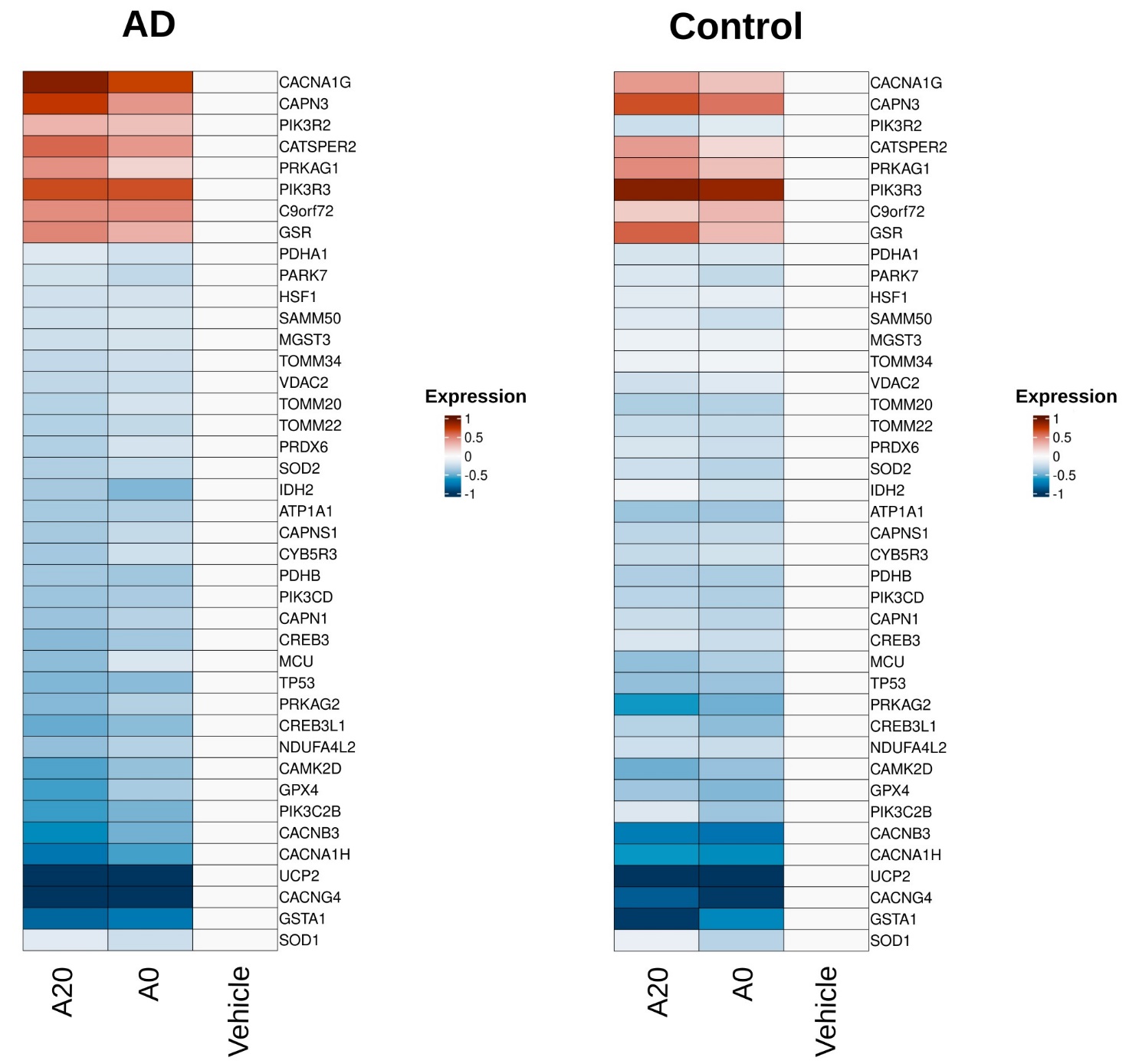


Heatmaps illustrating the mean expression levels of differentially expressed genes (DEGs) from IPA^®^ pathway mitochondrial dysfunction (excluding genes that are involved in the OXPHOS pathway), upon a 24-h exposure to the vehicle, A0, and A20. DEGs are centered around the average expression of the vehicle, within both AD and cognitively healthy control sample sets.

**Supplementary Figure 4. NADP concentrations in the 24-h vehicle and A20 exposed control and AD OM cells.**


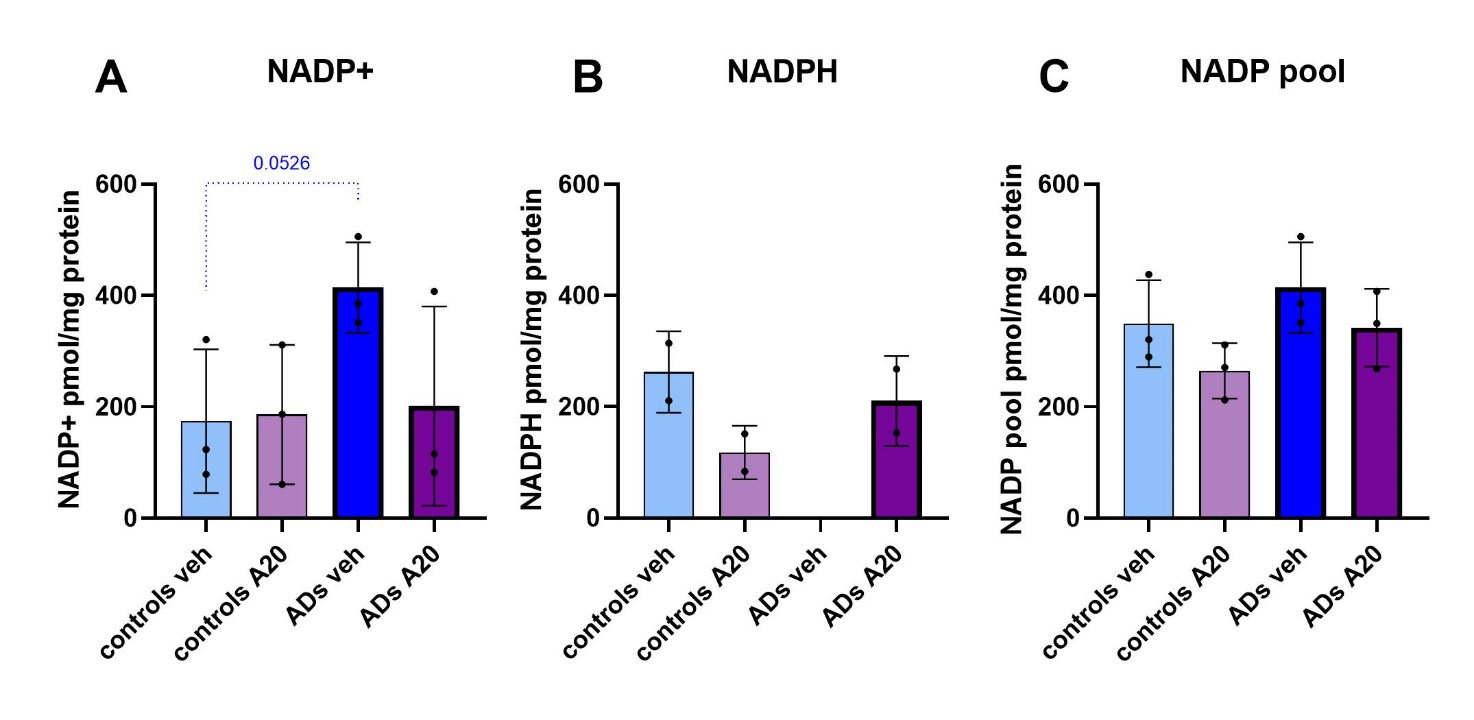


(A) NADP+ concentration is slightly elevated in ADs veh compared to all other treatments. However, when comparing controls veh to ADs veh with Student’s t-test, the difference does not quite reach the statistical significance (p<0.0526). (B) NADPH was detected only in a few samples (n=2/group), and not a single value was observed from ADs exposed to the vehicle. Yet it was interesting to discover that the same few samples where NADPH was detected at all, had also lower NADP+ levels compared to others. (C) However, the pool of NADP (NADP+ and NADPH together) was close to the level of NADP+ measured in samples with non-detectable NADPH. Despite the efforts of concentrating the samples up to fourfold, NADPH remained to be non-detectable in some of the samples. Results may suggest that NADPH is very labile in these cells and very easily gets converted into NADP+.
